# Correction: Hu et al. Dietary Net Energy Concentration Affects Growth Performance, Carcass Traits, Intramuscular Fatty Acid Profile, and Cecal Microbiota of Pigs with Restricted Feed Allowance. *Animals* 2025, *15*, 3514

**DOI:** 10.3390/ani16111656

**Published:** 2026-05-29

**Authors:** Qinfeng Hu, Wanxin Xiang, Youzhi Pu, Yong Zhang, Pan Zhou, Tiande Zou, Zhengjun Xie, Zhiqing Wu, Xiang Ao, Jinming You, Honglin Yan

**Affiliations:** 1 College of Life Science and Agri-Forestry, Southwest University of Science and Technology, Mianyang 621010, China; qinfenghu02@163.com (Q.H.); 13438461953@163.com (W.X.); puyouzhi2023@163.com (Y.P.); zyzlrzjh@swust.edu.cn (Y.Z.); pamelazhoupanpan@aliyun.com (P.Z.); 2 Jiangxi Province Key Laboratory of Animal Nutrition, College of Animal Science and Technology, Jiangxi Agricultural University, Nanchang 330095, China; tiandezou@jxau.edu.cn; 3 Shuangbaotai Group Co., Ltd., Nanchang 330095, China; xiezj@sbtjt.com (Z.X.); yhy20000123@163.com (Z.W.); 4 Guangnan (Zhanjiang) Jiafeng Feed Co., Ltd., Zhanjiang 524033, China; 5 Chengdu Tieqilishi Feed Co., Ltd., Sichuan TQLS Group, Chengdu 610200, China; ao_sunshine@hotmail.com

**Error in Table**

In the original publication [1], there was a mistake in Table 4 as published. “First rib backfat thickness”, “Last rib backfat thickness”, “Lumbosacral junction backfat thickness”, and “Mean backfat thickness” were mistakenly labeled as “mm” instead of the correct “cm”; “Carcass weight” was mistakenly labeled as “g” instead of the correct “kg”. All other units in the article are consistent and accurate. The corrected Table 4 appears below. The authors state that the scientific conclusions are unaffected. This correction was approved by the Academic Editor. The original publication has also been updated.

## Figures and Tables

**Table 4:** Effects of dietary energy density on carcass traits of pigs with restricted feed allowance.

Items	CON ^1^	Dietary Energy Density for Non-AD Pigs ^2^			SEM	*p*-Value		
		NE 2330	NE 2370	NE 2410		ANOVA	Linear	Quadratic
Carcass weight, kg	91.79 ^a^	82.78 ^c^	87.43 ^b^	88.51 ^ab^	1.23	<0.001	<0.001	0.582
Dressing percentage, %	75.72 ^a^	74.48 ^b^	74.85 ^b^	74.91 ^b^	0.15	<0.001	<0.001	0.149
First rib backfat thickness, cm	5.55 ^a^	4.31 ^c^	4.82 ^b^	4.95 ^b^	0.15	<0.001	<0.001	0.775
Last rib backfat thickness, cm	3.40 ^a^	2.44 ^b^	2.77 ^b^	2.92 ^ab^	0.21	0.028	0.004	0.728
Lumbosacral junction backfat thickness, cm	3.60 ^a^	2.44 ^b^	3.01 ^b^	3.11 ^b^	0.18	<0.001	<0.001	0.804
Mean backfat thickness, cm	4.18 ^a^	3.06 ^c^	3.53 ^b^	3.66 ^b^	0.12	<0.001	<0.001	0.836
Loin muscle area, cm^2^	35.04 ^a^	32.64 ^b^	34.17 ^ab^	35.31 ^a^	0.72	0.060	0.015	0.225

Results are presented as mean and SEM (*n* = 8). ^a,b,c^ Within a row with different superscripts differ significantly (*p* < 0.05). ^1^ CON, basal diet. ^2^ AD, ad libitum feeding.
